# Dental Education

**Published:** 1864-04

**Authors:** J. Taft


THE
DENTAL REGISTER OF THE WEST.

VOL. XVIII.] APRIL, 1864. [No. 4.

### Original Essays and Communications.

### DENTAL EDUCATION.

BY J. TAFT.

Though the subject of dental education may be regarded as an old and hackneyed one, it is nevertheless one of great importance, laying just at, and indeed constituting the foundation of what we regard as a noble profession, without which a permant comely structure can not be reared.

I do not now propose to discuss professional education, but that which preceeds it, or should do so; viz: general literary and scientific attainments, together with, habits of thought, study, research and investigation.

Now what are the admissions, facts compel us to make in reference to this matter.

And in the first place we must admit that a very large proportion, of those now in the profession, had not at the time of their entering it, a respectable English education, to say nothing of scientific attainments; and but few, have made much, if any progress, since that time, or ever will.

And again, many of those now entering the profession, are sadly deficient in elementary attainments; and they are necessarily without habits of systematic thought or study, and hence have not that mental vigor and force, that characterizes the educated man, and the student.

Many things have been proposed for professional improvement and advancement; most of which are good, in themselves; but many of them impracticable, because of the inability of men to employ them.

The practical question for us to consider is, how shall this difficulty be remedied, and the desired condition brought about.

When circumstances were such, that men too indolent to engage in other pursuits,—supposing this to be one without labor,—and desirous of obtaining money rapidly with little effort, supplied themselves with a few tools, and sometimes a book or two; entered the practice of the profession, as they supposed fully armed and equipped; without so much as saying by your permission to anybody; nothing less could be expected than that the most illiterate and ignorant would press into the ranks of the profession; and thus preclude, to a large extent, those more worthy than themselves.

But this state of things is now undergoing a change, a professional wall is now being built up, in the shape of Dental Colleges and Associations, and we hope it will be built so high and strong, that non can scale or break it down, but that all who enter will be compelled to do so through the legitimate and will guarded gate-ways.

Associations have during the last ten years operated with considerable power upon many in the profession, in reference to the selection of those whom they would initiate into our ranks. Many have fully concurred in and operated upon resolutions requiring them to receive no students for a less time than two and in some instances three years. Such regulations, so far as we know, have only had reference to *time* of studentship; leaving out of view, and indeed making no suggestions as to the preliminary qualifications of the student. We think there is room here for accomplishing something, at least suggestively, if not otherwise. Associations should take this matter in hand and make such provisions as would meet the difficulty.

Our associations will ere long, embrace all the best members of the profession; and they converging to, and operating through a common center, will possess, and be able to exercise an irresistible influence in all such matters.

Our Dental Colleges have not, we are sorry to say, accomplished as much in this direction as they might, nor as much, indeed, as some of our Dental Societies; we know of no Dental College, as yet, where preliminary examinations are made and a thorough English education even insisted upon; neither do we know of any that makes a private pupilage, or prior dental studies, a requisite. Those of but little literary, and no professional attainments are admitted to our Dental Colleges, and pressed on through two courses of lectures and demonstrations, to an examination for graduation; now at this point, a large proportion of the theses presented to the faculties of our colleges, will exhibit a gross deficiency in the knowledge of the writers, of the structure of language, to say nothing further, and how much greater the deficiency would appear if an investigation was pressed in other directions, we will not now attempt to define. That there is a deplorable defect here, no one will pretend to deny.

Now what is the remedy to be applied, and who shall begin the work? We know some noble men in the profession, who will not receive a student unless he has the requisite qualifications, but they are few.

We would suggest that our dental associations at once go to work in this matter; they can do much, indeed they are *the* power in the profession; and we think it quite time that they begin to guard themselves against improper intrusions.

Let them imbue their members with such a zeal, that they will not assist, or countenance the approach to our ranks, of those who are without ample preliminary qualifications.

Our Colleges should at once, without fear or favor, institute preliminary examinations, and admit no student to their halls, who can not stand a thorough test.

Let all the Colleges adopt an uniform course in this matter, and there will be no difficulty; and especially will it be easy, if they have the concurrence and coöperation of the Associations. But it is said the classes will be small; be it so, better far to have a *few* good, than many indifferent and bad; but though that might at first, it would not long be the case, for as the classes would be made better, a better class of men would seek to enter them, and thus elevation would necessarily follow.

We hope this subject will be taken up and discussed by others, till desirable results are obtained.

